# Efficacy of Vortioxetine Versus Escitalopram on the Cognitive Profile of Patients With Depressive Disorder: A Comparative Study

**DOI:** 10.7759/cureus.79365

**Published:** 2025-02-20

**Authors:** Saloni Mishra, Varchasvi Mudgal, Koustubh R Bagul, Priyash Jain, Virendra Pal

**Affiliations:** 1 Psychiatry, Mahatma Gandhi Memorial Medical College, Indore, IND

**Keywords:** cognition, depression, depressive disorders, escitalopram, vortioxetine

## Abstract

Background: Major depressive disorder (MDD) is a prevalent mental health condition with significant cognitive impairments, affecting millions worldwide. Cognitive dysfunction in MDD, encompassing attention, memory, executive function, and processing speed deficits, increases the disease burden and impacts treatment outcomes. Vortioxetine, a multimodal antidepressant, and escitalopram, a selective serotonin reuptake inhibitor, have both been utilized for MDD treatment, yet their comparative effects on cognitive function remain under-explored.

Aim: This study aimed to compare the effects of vortioxetine and escitalopram on cognitive function in patients with MDD.

Methodology: This prospective, randomized follow-up study (October 2023-2024) assessed 150 MDD patients meeting the following criteria: Montreal Cognitive Assessment Scale (MoCA) score ≤ 26 and Brief Cognitive Rating Scale (BCRS) score ≥ 1. Patients were randomly assigned to escitalopram (10 mg, n = 78) or vortioxetine (10 mg, n = 72), with 50 per group analyzed at the final evaluation. Cognitive function was assessed using MoCA and BCRS at baseline, week two, and week four.

Results: Both vortioxetine and escitalopram improved cognitive function over four weeks. While the reduction in BCRS scores showed no statistically significant difference between the groups, MoCA scores indicated a slight advantage for escitalopram by the fourth week (p = 0.05). These findings suggest that both drugs effectively improve cognitive function, with escitalopram demonstrating a slight cognitive advantage over the study period.

Conclusion: Both vortioxetine and escitalopram improve cognitive symptoms in MDD, with escitalopram showing a modest cognitive advantage by the 4th week. These results support the efficacy of both medications for cognitive symptoms in MDD, with escitalopram potentially offering a slight edge in cognitive enhancement. Further long-term studies are warranted to confirm these findings and investigate the underlying neurobiological mechanisms.

## Introduction

Depression is a widespread mental health condition that affects individuals of all ages and genders in India and around the world. Globally, depressive disorder was recognized as the third most common cause of disability in 2015 [1]. In India, the National Mental Health Survey 2015-16 found that nearly 15% of Indian adults need active intervention for one or more mental health issues and one in 20 Indians suffers from depression [2]. The incidence of depression in India was estimated to be 4.5%, which comes around to 56 million [3]. By 2030, unipolar depression is expected to become the second-largest contributor to the global burden of disease and thus has a huge impact on individuals, families, and societies [4]. Cognitive dysfunction in depression encompasses deficits in attention, memory, executive function, and processing speed, among others. These impairments contribute to the overall burden of the illness and impact treatment outcomes and functional recovery. Therefore, understanding how antidepressants affect cognitive function is crucial for optimizing treatment strategies and improving patient outcomes [5].

Vortioxetine is a novel drug for depression with a multimodal mechanism of action, acting both as an inhibitor of the serotonin (5-HT) transporter as well as a modulator of several 5-HT receptor subtypes (5-HT3, 5-HT7, and 5-HT1D receptor antagonist, 5-HT1B receptor partial agonist, and 5-HT1A receptor agonist). Vortioxetine is effective and well-tolerated for the treatment of depressive, cognitive, and physical symptoms in patients with major depressive disorder (MDD) across the approved dose range of 5-20 mg/day [6].

A study by Katona et al. [7] in older patients with MDD found that vortioxetine and duloxetine had major effects on verbal learning and memory, compared with a placebo. Still, only vortioxetine significantly affected a test of processing speed and executive function. Results of a large clinical trial by McIntyre et al. evaluating the consequential of vortioxetine, compared with placebo, on measures of cognitive dysfunction as a primary endpoint in younger individuals with depressive disorder support the beneficial effects of this medication on objective and subjective measures of cognitive function [8]. In contrast, escitalopram, while effective in alleviating depressive symptoms, has shown limited evidence of cognitive enhancement [9]. As a traditional selective serotonin reuptake inhibitor (SSRI), escitalopram primarily targets serotonin reuptake inhibition, with fewer effects on other neurotransmitter systems implicated in cognitive function [10]. While some studies have reported modest improvements in cognitive function with escitalopram treatment, the evidence remains inconsistent, and the extent of its cognitive effects relative to vortioxetine is not well-established.

Escitalopram was chosen for comparison because it is one of the most commonly prescribed SSRIs for MDD. While it is effective in alleviating depressive symptoms, its impact on cognitive function remains inconsistent in existing studies, as mentioned above.

Vortioxetine, on the other hand, has been shown to have cognitive-enhancing properties due to its multimodal action on serotonin receptors. Comparing vortioxetine with escitalopram helps determine whether vortioxetine offers superior cognitive benefits over a well-established SSRI, providing valuable insights for optimizing antidepressant treatment, especially in patients with cognitive impairment.

This study aims to evaluate whether vortioxetine or escitalopram produces greater cognitive improvement in MDD patients over four weeks, as measured by the Montreal Cognitive Assessment Scale (MoCA) and Brief Cognitive Rating Scale (BCRS) scores.

## Materials and methods

**Figure 1:**
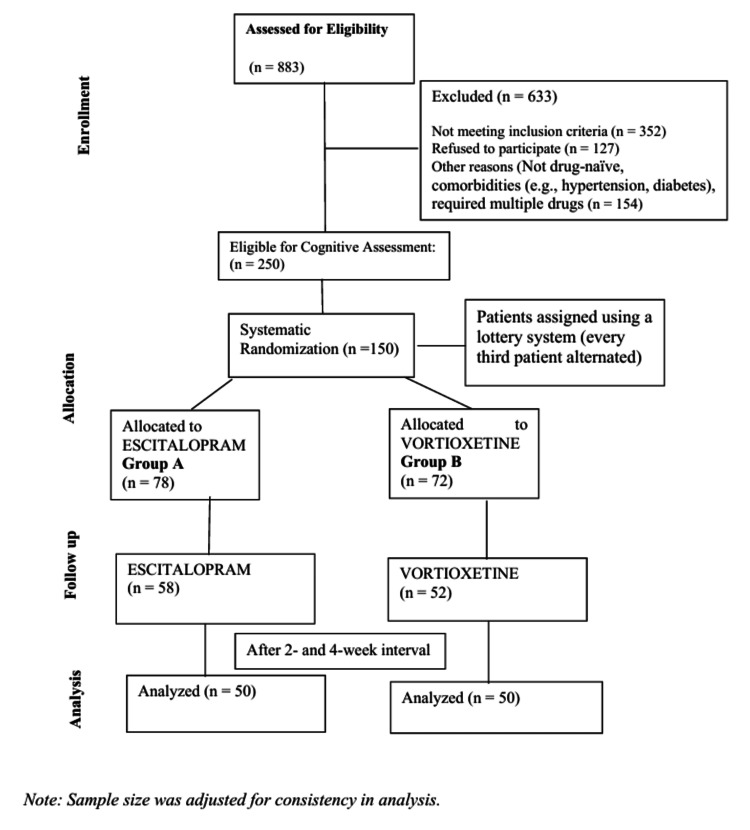
Consolidated Standards of Reporting Trials (CONSORT) diagram. Diagrammatic representation of the methodology.

This prospective randomized, comparative follow-up study was conducted from October 2023 to October 2024 at Maharaja Yeshwantrao (MY) Hospital, Indore. A total of 883 patients were assessed for a depressive episode, out of which 633 were excluded for various reasons, such as requiring multiple medications, not being drug-naïve, or having comorbidities like hypertension and diabetes. The remaining 250 patients were evaluated using the MoCA and BCRS for cognitive impairment and a further 150 patients were found to be having cognitive impairment. These 150 patients were randomly assigned to two treatment groups (group A and group B) using a lottery system, where every third patient was assigned alternately. Group A (n = 78) received escitalopram (10 mg), while group B (n = 72) received vortioxetine (10 mg), with no additional treatment modalities (Figure 1).

By the final evaluation, 58 patients remained in the escitalopram group and 52 in the vortioxetine group. For consistency and ease of analysis, the sample size was adjusted to 50 patients per group in the final statistical comparisons.

The doses of vortioxetine (10 mg) and escitalopram (10 mg) were chosen for their established efficacy, tolerability, and real-world relevance. Keeping both at 10 mg ensures a fair comparison while minimizing dose-dependent variability and side effects. This approach aligns with prior research assessing cognitive effects in MDD. This restriction ensures a balanced, practical, and clinically relevant evaluation of cognitive effects.

Enrollment ceased once 50 patients had been included in each treatment arm. Only 50 patients per group were included to ensure a manageable sample size while maintaining statistical power. The remaining patients were excluded due to factors like dropouts, non-adherence, side effects, or loss of follow-up.

Participants were recruited from a tertiary care center's outpatient department (OPD) and selected based on specific inclusion and exclusion criteria. The inclusion criteria included newly diagnosed depression according to the International Classification of Diseases, Tenth Revision (ICD-10), age between 18 and 65 years, drug-naïve, a MoCA score ≤ 26, and a BCRS score ≥ 1. The exclusion criteria included concurrent use of cognition-impairing medication like drugs with potential anticholinergic action like tricyclic antidepressants, antipsychotics, benzodiazepines, etc., and history of trauma, substance use, and pregnancy. Assessments were done using MoCA [11] and BCRS [12] at the baseline, 2nd week, and 4th week. The study was conducted from October 2023 to October 2024 (one year) for patient recruitment and data collection. However, individual patient follow-up was limited to four weeks. This design allowed sequential enrollment over a year but restricted outcome assessments to short-term effects. Descriptive statistics and tests of significance were used to analyze the data.

The institution's research ethics committee approved the research plan on 11th September 2023 (IEC- EC/MGM/Sept-23/66). Data used in the study were anonymized for confidentiality, and written consent was obtained from all participants.

As this clinical trial was an academic trial, according to rule 2(a), CT 2019, “CT rules are not applicable on an academic clinical trial of a drug already approved for a certain claim and initiated by any investigator, academic or research institution for a new indication or new route of administration or new dose or new dosage form, where the results of such a trial are intended to be used only for academic research purposes and not for seeking approval of the CLA or regulatory authority of any country for marketing or commercial purposes” [13].

## Results

For the final evaluation, 58 patients remained in the escitalopram group (group A) and 52 in the vortioxetine group (group B). For consistency and ease of analysis, the sample size was adjusted to 50 patients per group in the final statistical comparisons.

As shown in Table 1, participants in the escitalopram group (group A) had a mean age of 43 ± 13 years, while the vortioxetine group (group B) had a mean age of 40 ± 13 years, establishing a balanced age distribution across groups. Males predominated in both groups, constituting 76% (38) in group A and 60% (30) in group B. Marital status analysis revealed that married individuals were more prevalent, at 68% (34) in group A and 62% (31) in group B, indicating a higher treatment-seeking rate among married individuals.

**Table 1:** Demographic profile.

Age group (years)	Group A (escitalopram) (N = 50)	Group B (vortioxetine) (N = 50)
21-30	15	13
31-40	8	16
41-50	6	6
51-60	19	12
61-70	2	3
Mean ± SD (age)	43 ± 13	40 ± 13
Sex		
Male	38	30
Female	12	20
Marital status		
Married	34	31
Unmarried	16	19

The statistical analysis of demographic variables showed no significant differences between the two groups. The t-test for age distribution yielded p = 0.251, indicating a similarity in age between the escitalopram and vortioxetine groups. The chi-square test for sex distribution (p = 0.133) and marital status (p = 0.675) also showed no significant differences.

The BCRS score differences between escitalopram (group A) and vortioxetine (group B) were not statistically significant at any time point (p > 0.05). Although there was a trend toward a greater reduction in BCRS scores for escitalopram by the 4th week, this did not reach statistical significance.

At baseline and the 2nd week, the differences in MoCA scores between the two groups were not statistically significant (p > 0.05). By the 4th week, however, the difference reached the threshold of statistical significance (t = 2.07, p = 0.05), indicating that escitalopram might have a more favorable effect on cognitive function compared to vortioxetine (Tables 2, 3).

**Table 2:** Comparison between BCRS and MoCA scores of escitalopram and vortioxetine at 0, 2nd, and 4th week. BCRS: Brief Cognitive Rating Scale; MoCA: Montreal Cognitive Assessment Scale.

Variable	Group (n = 50 each)	Mean ± SD	t-value	p-value (≤0.05)
BCRS 0 week	Group A	2.48 ± 1.05	0.19	0.84
	Group B	2.44 ± 0.99		
BCRS 2nd week	Group A	2.04 ± 1.02	-0.39	0.69
	Group B	2.12 ± 1.00		
BCRS 4th week	Group A	1.50 ± 0.73	-1.59	0.11
	Group B	1.78 ± 0.99		
MOCA 0 week	Group A	24.18 ± 2.15	0.38	0.70
	Group B	24.02 ± 2.01		
MOCA 2nd week	Group A	25.24 ± 1.91	1.42	0.15
	Group B	24.68 ± 2.01		
MOCA 4th week	Group A	26.04 ± 2.04	2.07	0.05
	Group B	25.22 ± 1.90		

**Table 3:** Test of significance for change in cognitive deficit. BCRS: Brief Cognitive Rating Scale; MoCA: Montreal Cognitive Assessment Scale.

Variable	Drug	Improved	Not improved	p-value (≤0.05)
MOCA	Group A	30	20	0.36
	Group B	27	23	
BCRS	Group A	32	18	1.03
	Group B	27	23	

The chi-square test results indicate no statistically significant difference in cognitive improvement between patients treated with escitalopram and those treated with vortioxetine when assessed using both the MOCA and BCRS scores.

## Discussion

The present study was conducted at the Department of Psychiatry, Mahatma Gandhi Memorial Medical College and Mental Hospital, Indore, to compare the effects of vortioxetine vs. escitalopram on the cognitive profile of patients with MDD. This prospective, hospital-based follow-up study utilized the BCRS and the MoCA to evaluate cognitive function in patients with depressive episodes over a four-week period.

The primary aim of this study was to determine whether vortioxetine or escitalopram provides greater cognitive improvement in patients with MDD. Both medications led to cognitive improvement over the study period. However, while reductions in BCRS scores did not show significant differences between the two drugs, MoCA scores indicated a slight cognitive advantage for escitalopram by the fourth week (p = 0.05). These findings suggest that escitalopram may offer a modest benefit in enhancing cognitive function within the early stages of treatment.

In group A (escitalopram), the mean age was 43 ± 13 years, while in group B (vortioxetine), it was 40 ± 13 years, ensuring a comparable age distribution. Males predominated in both groups (76% (38) in group A, and 60% (30) in group B), suggesting a higher likelihood of men seeking treatment in this setting, despite depression being more prevalent in women. Married individuals were also more common (68% (34) in group A, and 62% (31) in group B), possibly due to stronger social support encouraging treatment. Prior studies highlight the protective role of social and relational factors in depression recovery [14,15].

None of the demographic variables (age, sex, marital status) showed a statistically significant difference (p > 0.05; p > 0.05; p > 0.05), and confounding due to these factors is unlikely in this study. Therefore, the observed differences in cognitive function between escitalopram and vortioxetine are likely due to the medication effects rather than demographic biases.

Both medications showed a reduction in BCRS scores over time, indicating improved cognitive function. However, escitalopram appeared to have a slightly better outcome by the 4th week, with a lower mean score (1.50) than vortioxetine (1.78). The lower BCRS score for escitalopram suggests it may be more effective in improving cognitive symptoms of depression within the first month of treatment. Similar findings were reported by Huang et al., who assessed vortioxetine and escitalopram regarding cognition and depressive symptoms, noting numerical improvements across the Digit Symbol Substitution Test (DSST), Perceived Deficits Questionnaire (PDQ), and Montgomery-Åsberg Depression Rating Scale (MADRS) scores, although these results were not statistically significant [16]. A study by Rapaport et al. [17] found that escitalopram was effective in improving cognitive performance even after a relatively short treatment period. Additionally, a study by Savaskan et al. demonstrated that escitalopram is effective in treating depression in elderly patients and may improve cognitive performance related to social stimuli [18].

Both medications also showed an increase in MoCA scores over time, indicating cognitive improvement. Escitalopram again demonstrated a slightly superior outcome, with a higher mean score (26.04) than vortioxetine (25.22) by the 4th week. This suggests that escitalopram may have a more pronounced positive effect on cognitive function in depressed patients over the first month of treatment, as supported by other studies like that of Savaskan et al. [18].

The chi-square test results indicate no statistically significant difference in cognitive improvement between the escitalopram and vortioxetine groups when assessed using the MoCA and BCRS scales. Several factors may account for this finding. Both medications are established antidepressants with known efficacy in cognitive improvement, potentially minimizing measurable differences within the short study duration. The sample size may have also been insufficient to detect subtle cognitive differences between the two drugs, limiting the statistical power of the analysis. Additionally, while MoCA and BCRS are widely used cognitive assessment tools, they may lack the sensitivity required to detect nuanced cognitive changes, particularly within a brief follow-up period. Furthermore, cognitive enhancement in MDD is closely linked to mood improvement, and since both medications effectively alleviate depressive symptoms, their cognitive effects may appear similar in the early stages of treatment.

Cohen’s d-effect size was calculated to assess the clinical relevance of the cognitive improvement between the two groups. The effect size for the difference in MoCA scores between escitalopram and vortioxetine was 0.42, indicating a small to moderate effect. While statistically significant, this suggests that escitalopram provides only a modest cognitive advantage over vortioxetine. While the difference is statistically significant, the clinical utility is modest, indicating that escitalopram provides a slight cognitive advantage over vortioxetine, but the real-world impact may not be substantial.

Despite the overall similarity, escitalopram demonstrated a marginally greater improvement in cognitive function (BCRS and MoCA scores) over four weeks compared to vortioxetine. This finding aligns with some existing literature suggesting escitalopram’s superior efficacy in alleviating cognitive impairments associated with depression. For instance, a study by Ali and Lam (2011) [19] highlighted the cognitive benefits of escitalopram in the treatment of depression. However, this contrasts with previous research suggesting that vortioxetine, due to its multimodal mechanism of action, may offer superior cognitive benefits [6]. Vortioxetine has previously demonstrated direct effects on objective measures of cognition in three large-scale, pivotal studies of depressed patients [7,8,20]. Consistent across studies, improvements seen in cognitive performance on the DSST were not accounted for by improvements in mood symptoms. This supports the specificity of vortioxetine on cognitive symptomatology in MDD, which may differentiate vortioxetine from other types of antidepressant pharmacotherapies [21]. Vortioxetine has previously been shown to improve a patient’s performance-based functional capacity as measured by the University of California, San Diego Performance-Based Skills Assessment Brief (UPSA-B) [20]. The results from this study, showing that improvements in cognitive function were associated with functional improvements, highlight the importance of addressing cognitive symptoms for restoring functional status in patients with MDD. Another network meta-analysis assessed the effects of antidepressants on cognitive dysfunction in MDD, focusing on the DSST [22]. Among 72 randomized controlled trials, vortioxetine showed a significant cognitive benefit over placebo (standardized mean difference = 0.325, p = 0.009) and was superior to escitalopram, nortriptyline, SSRIs, and tricyclic antidepressants. Other antidepressants, like duloxetine and sertraline, showed improvements but lacked statistical significance. The study highlights vortioxetine’s potential cognitive benefits, emphasizing the need for further research.

A potential explanation for this discrepancy lies in the study’s short duration. Many studies reporting the cognitive advantages of vortioxetine have assessed patients over extended periods, implying that its effects may take longer to manifest compared to escitalopram, which, as an SSRI, may facilitate faster cognitive improvements through early mood stabilization.

Several limitations must be considered when interpreting these findings. The relatively small sample size may have limited the study’s ability to detect statistically significant differences between the treatment groups. The four-week follow-up period may not have been adequate to capture the full extent of vortioxetine’s cognitive benefits. Additionally, baseline differences in cognitive function between groups could have influenced the observed outcomes, potentially exaggerating the effect of one drug over the other. The absence of a placebo-controlled group further restricts the ability to determine whether the cognitive improvements observed were solely attributable to the medications or influenced by external factors, such as repeated cognitive testing. Furthermore, unmeasured confounding variables, including medication adherence, comorbid conditions, and lifestyle factors, may have played a role in the results. Future research with larger sample sizes, extended follow-up periods, and additional cognitive assessment measures with neuroimaging and genetic analysis will be essential to provide a clearer and more comprehensive comparison of escitalopram and vortioxetine’s effects on cognitive function in MDD.

## Conclusions

Given that cognitive impairment is a critical aspect of MDD, the choice of treatment may consider specific cognitive benefits. Although vortioxetine showed slightly higher improvements in cognitive scores, the lack of statistical significance suggests that escitalopram remains a viable option, particularly when considering other factors such as patient response and side effect profiles.

Both escitalopram and vortioxetine showed efficacy in improving cognitive function in MDD patients. Escitalopram demonstrated a slight advantage in MoCA scores, suggesting its potential for cognitive enhancement. However, the lack of significant differences indicates that both drugs can be effective choices for treating cognitive symptoms of depression.
